# Efficiency of Ontario primary care physicians across payment models: a stochastic frontier analysis

**DOI:** 10.1186/s13561-016-0101-y

**Published:** 2016-06-06

**Authors:** Maude Laberge, Walter P. Wodchis, Jan Barnsley, Audrey Laporte

**Affiliations:** 1 Department of Health Services Research, Management and Policy, College of Public Health and Health Professions, University of Florida, 1225 Center Dr, Room 3111, Gainesville, FL 32610 USA; 2 Canadian Centre for Health Economics, Toronto, Canada; 3 Institute of Health Policy, Management, and Evaluation, University of Toronto, Toronto, Canada; 4 Institute for Clinical Evaluative Sciences, Toronto, Canada; 5 Toronto Rehabilitation Institute, Toronto, Canada

**Keywords:** Efficiency, Physician remuneration, Frontier analysis, Primary care, Productivity

## Abstract

**Objective:**

The study examines the relationship between the primary care model that a physician belongs to and the efficiency of the primary care physician in Ontario, Canada.

**Methods:**

Survey data were collected from 183 self-selected physicians and linked to administrative databases to capture the provision of services to the patients served for the 12 month period ending June 30, 2013, and the characteristics of the patients at the beginning of the study period. Two stochastic frontier regression models were used to estimate efficiency scores and parameters for two separate outputs: the number of distinct patients seen and the number of visits.

**Results:**

Because of missing data, only 165 physicians were included in the analyses. The average efficiency was 0.72 for both outputs with scores varying from 4 % to 93 % for the visits and 5 % to 94 % for the number of patients seen. We observed that there were both very low and very high efficiency scores within each model. These variations were larger than variations in average scores across models.

## Background

Concerns over growing health care expenditures have led to interest in increasing the efficiency of health care providers, particularly in developed countries such as Canada [1]. Canada has a single-payer, publicly financed, universal health insurance system whereby all medically necessary services are provided free at the point of service. The system is highly decentralized with health care being a provincial jurisdiction. The majority of primary care has been delivered by primary care physicians in solo or group practices who were traditionally remunerated on a fee-for-service basis [2]. Since the early 2000s, there has been a shift in the organization and payment of primary care physicians, with Ontario considered the province to have introduced the largest number of alternative payment structures [3].

Various factors can affect the efficiency of primary care physicians, including remuneration methods and the organizational characteristics of the primary care practice, as well as the nature of the outputs measured. Over the past fifteen years, primary care in Ontario has been transformed away from almost exclusively fee-for-service towards mixed payments and new models of organization with interdisciplinary teams. The mixed payment mechanisms for physicians combine capitation, fee-for-service and incentive payments for the delivery of preventive services. The new primary care models also feature group and interdisciplinary teams, enrollment of patients with the physicians, and after-hours access requirements.

For the purpose of this study, Ontario primary care models are categorized as: Fee-for-Service (FFS); Family Health Group (FHG); blended capitation; Family Health Teams (FHTs); and salaried models. All but the FFS and salaried models include incentives to enroll patients and have requirements for after-hours care. Salaried physicians also practice in an interdisciplinary team-based environment.

The FFS model is defined by remuneration of physicians for each service provided, as determined by the schedule of benefits (SoB). FHG physicians are also remunerated mostly on a FFS basis, but a FHG must include at least three physicians. FHG physicians have incentives to enroll their patients: they receive a payment for each enrolled patient and bonus payments for achieving targets on cancer screening and chronic disease management for eligible enrolled patients. They also have requirements for after-hours care, i.e., that the clinic be opened on evenings and weekend days. In this study, FFS physicians are separated from FHG physicians because of these structural and payment differences.

Capitated physicians work in groups with a minimum of three physicians. The capitation rate covers a basket of primary care services and is adjusted for age and sex of patients. Physicians are remunerated through FFS for other services and they receive 15 % of the fees for services included in the capitation rate. It is estimated that about 60 % of their revenues come from capitation payments [4].

A FHT is a group of capitated physicians who receive supplemental funding from the Ontario Ministry of Health and Long Term Care (MOHLTC) to hire additional health care providers (such as nurses, social workers, dietitians, and pharmacists) and to create an interdisciplinary team. The choice of providers is meant to reflect the needs of the community served by the FHT.

Efficiency can be defined as the relationship between the observed ratio of outputs to inputs of a unit (such as a physician), compared to an optimal ratio. The optimal ratio is defined by the maximum output that could be produced with the same quantity of inputs or to the fewest inputs that could be used to produce the same level of output [5, 6]. Hence, efficiency depends on both the outputs and inputs chosen. Stochastic frontier analysis (SFA) is the most commonly used regression based approach to efficiency measurement.

The purpose of the present study is to identify the effect of the primary care model on a physician’s efficiency. The first part of the paper provides the framework for the measurement of efficiency. This is followed by the identification of the data and the design of the study. We then provide the results including a description of the characteristics of physicians and patients across models, the efficiency scores of physicians and the estimates of the effect of primary care models on efficiency. Finally, the paper discusses the implications of the results for health care policy.

### Payments mechanisms, physician behavior and a framework to measure efficiency

Payers have experimented with various methods to pay physicians, each method having incentives that could affect physicians’ behaviors in different directions.

Physicians paid on a FFS-basis have an incentive to provide more visits [7, 8] and this may be at the expense of conducting out-of-visit activities for which they do not receive any remuneration. Incentives to increase volumes was found to lead physicians to increase what Gaynor & Pauly termed “effort” [9], i.e. working longer hours, yet without improving efficiency. FFS physicians may also shorten the duration of consultations [10], leading to what some call the “one problem per visit” policy [11]. Hence, physicians receiving payment through FFS are expected to produce more services than physicians paid by blended capitation or salary. A review of payment methods also found that FFS physicians were more likely to provide elective procedures, supporting the idea of over-supply of services [12]. Their efficiency, however, may be similar to physicians in other payment methods, when considering the actual number of hours spent on direct patient care and controlling for the duration of the visits and other factors that could affect the production of services.

Physicians paid on a capitation basis have a different incentive: rather than maximizing services, their incentive is to maximize the number of patients enrolled with them. The capitation rates in Ontario are reduced for each patient beyond 2,400, meaning that there is diminishing marginal returns at that point. If physicians were trying to maximize profits, they would enroll patients until the marginal revenue no longer exceeds to marginal cost of treating a patient. To take on larger panel sizes, physicians may shift care by making more frequent referrals to specialists [13–17]. But they also have an incentive to find efficiencies within their organizational practices by working with other health care providers and using them as substitutes [18]. Under capitation, physicians receive the same monthly capitation payment per patient regardless of the number of visits a patient makes. If payments are not fully adjusted for the expected health care needs of the patients, physicians may “cherry-pick” patients expected to have a lower utilization than the capitation rate accounts for [17]. The Ontario capitation rates are only age and sex adjusted, which was found to lead to over-compensation for healthier and wealthier patients and under-compensation for patients of lower income and higher morbidity burden [19]. However, the two Ontario primary care models that use capitation as their basis for physician remuneration also include FFS and performance incentives, which were intended to reduce the “pathologies” associated with prospective payment [20] and could lead to higher efficiency.

Salary payment is seen as a disincentive to productivity [20]. In Ontario, salary payment was associated with lower productivity but higher quality of care [21, 22]. However, in the UK, a switch towards salary did not affect physicians’ productivity or quality of care compared to physicians paid on FFS or on capitation [23]. A review of empirical studies on payment methods suggests that the effect of salary on physician behavior is not conclusive [24] while another review found that patients of salaried physicians were more satisfied with access compared to those of FFS physicians [8].

The measure of efficiency relies on determining the relationships between inputs and outputs, and their selection generally depends on the context and the perspective taken. A private primary care organization may be interested in the allocation of its resources to optimize outputs. As the organization pays for the remuneration of physicians, other employees, as well as office space and equipment, it considers all of these inputs in the production of services. Using the revenues generated as the measure of outputs in relation to the costs of delivering services would be aligned with an aim of maximizing profits [25–27].

From the perspective of a public health care insurance provider such as the Ontario government, the aim is to improve access to health care services by the population. Outputs include the number of primary care visits and the number of people served, which are both commonly used, in studies examining productivity and efficiency of primary care providers [21, 28–31]. In Ontario, the remuneration of primary care physicians is meant to cover the operational costs (rent, administrative staff, etc.) of delivering services as well as physician time. The mix of administrative staff and other types of health care providers in a practice is also highly linked to the primary care model. For example, most salaried physicians worked in an interdisciplinary environment that employs registered nurses and nurse practitioners and so do FHTs.

**Fig. 1:**
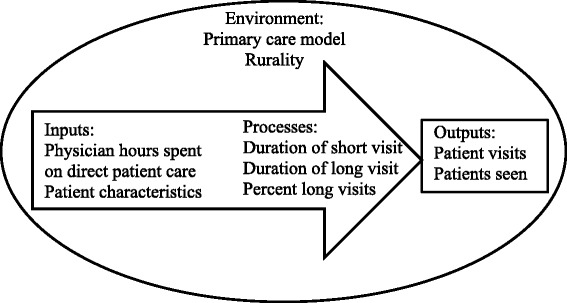
Conceptual Framework of the Primary Care Physicians Production of Services

Figure 1 presents the conceptual framework applied in the present study. It considers the number of hours that physician spend on direct patient care as the input in the production of services.

Health care services in general are very labor intensive which is aligned with the use of physicians as the main inputs in the primary care efficiency literature [21, 27, 32–34]. The measure of physician input varies across studies and appears to simply reflect availability of data in choosing a full-time equivalent (FTE) count or the number of weekly hours worked; the latter being more precise, but neither specifying the time spent on direct patient care. Both these approaches assume that all of a physician’s work time is spent on direct patient care. Yet primary care physicians spend over 20 % of their workday on patient care activities outside of office visits, which in part substitute for visits, and play an important role in the coordination of care [35, 36]. Because the outputs from the time spent on these activities cannot be measured, it is logical to exclude that time from the input as well. In addition, the time that physicians spend on such activities may vary across payment models.

The environment and the practice style of a physician may also affect the outputs. The environment is considered here as an organizational environment with the primary care model and the geographic location, with rurality. How a physician works in terms of the durations of short and long visits and the percentage of long visits are considered process measures. In addition to the practice characteristics, patient characteristics such as specific diseases and the number of chronic diseases were found to affect the production of services [37].

## Methods

### Data and variables

The sample for the study included all 183 physician respondents to the Quality and Costs of Primary Care (QUALICOPC) study in Ontario. Data were collected between January and July 2013. QUALICOPC is an international study that included 34 countries. In each country, the same set of four surveys was used to collect data about practice characteristics, physician perceptions of care, patient perceptions of care, and patient values, with only minor changes to accommodate the specific realities of the local health care system [38, 39]. In Ontario, recruitment was conducted in collaboration with the Ontario College of Family Physicians. The College included a recruitment announcement in their newsletter that was sent to all primary care physicians in the province - over 13,000 [40]. Interested physicians contacted the research team at the University of Toronto and those meeting eligibility criteria were sent a survey package (*n* = 229). Of those, 183 physicians completed and returned the survey package. Only one physician per practice was eligible to participate in order to maximize the number of practices involved. There was a CAD200 incentive for participants to cover costs of disruption to the practice. The sample included physicians in an array of models including: FFS (10 %), FHG (22 %), FHT (31 %), blended capitation (30 %) and salaried (6 %). These groups were treated as mutually exclusive, and so the blended capitation group excluded physicians working in FHTs. In comparison, the distribution of family physicians in Ontario is currently as follows: 20.5 % in FHGs, 42 % in blended capitation, 22 % in FHTs, with the remaining in other models including salaried and FFS [41].

Data for this study included elements of the Practice and Physician Surveys from QUALICOPC linked with health administrative databases, and also census data held at the Institute for Clinical Evaluative Sciences (ICES) at the University of Toronto. QUALICOPC participants were asked to provide their Ontario Health Insurance Plan (OHIP) billing numbers, which were used to link to patient visits in the OHIP database. Based on the physician’s billing number, a dataset at the patient level was built, using each patient’s own ICES Key Number (IKN) to link with health care utilization. Utilization included both outpatient and inpatient from the OHIP database and hospital databases, including the Discharge Abstract Database (DAD) and the National Ambulatory Care Reporting System (NACRS). From this patient level dataset and data from the QUALICOPC survey, a physician level dataset was constructed for use in the SFA.

The present study considers two physician outputs that are common in the literature [21, 28–32, 34]: the number of distinct patients seen and the number of visits, from the OHIP database. The outputs were adjusted with the average duration of a long consultation, the average duration of a short consultation and the percentage of long consultations, reported by physicians in the QUALICOPC survey. The input was the number of weekly hours spent on direct patient care as reported by physicians in the QUALICOPC survey.

Patient characteristics included socio-economic status with the average income quintile from the Census database, average age, percentage of female patients, and health status. Health status was measured using the Johns Hopkins Adjusted Clinical Group® (ACG®) weight [42].

The analyses also adjusted for a rural location of the practice, which was reported by physicians in the survey.

### Analytical approach

This is a cross-sectional study assessing the efficiency of Ontario primary care physicians across different primary care models using the SFA method. With the SFA method, efficiency is determined in relation to a production frontier, which is itself based on the actual productivity of physicians observed in the study sample as measured in terms of both patients seen and number of visits respectively in each estimated efficiency model [43, 44]. This method has the benefit of assessing the effect of covariates on the efficiency scores [30, 45].

The SFA approach requires specifying a form of the production function, which is typically a Cobb-Douglas or a more general translog form. The error term *e*_*i*_ in the regression model for a producing unit *i* is composed of two components: inefficiency and noise: *e*_*i*_ *= u*_*i*_ *+ v*_*i*_ [46]. We used a one-step approach in which coefficients on explanatory variables and efficiency scores are estimated jointly. The explanatory variable set includes dummies corresponding to each of the primary care models as well as other variables on the level of output.

Because the Cobb-Douglas is easier to interpret and requires the estimation of fewer parameters, it is preferred to the translog. However, the translog is used when the assumption of unitary substitution elasticities required for the Cobb-Douglas does not hold. Both forms of the production function were generated. The test failed to reject the null hypothesis of a Cobb-Douglas model and the Cobb-Douglas was selected. Two distinct Cobb-Douglas production functions were estimated, one for each of the two outputs: the number of patients seen in one year and the number of visits in one year. Each physician’s productivity was determined by the quantities of inputs utilized and the volumes of outputs produced. Efficiency scores were then estimated for each physician. Input and output variables were log-transformed such that:

1 $$ \ln\ {\mathrm{y}}_{\mathrm{i}}=\upbeta ' \ln\ {\mathrm{x}}_{\mathrm{i}} + {\mathrm{v}}_{\mathrm{i}}\hbox{--}\ {\mathrm{u}}_{\mathrm{i}} $$

Where:

ln y_i_ is the logarithm of the output (the number of patients seen or the number of visits) by physician i; β’ is the vector of the parameters to be estimated; ln x_i_ is a vector of the logarithm of the inputs; v_i_ is statistical noise, which can be positive or negative and is assumed to follow a normal distribution centered at zero; u_i_ is productive inefficiency. Productive inefficiency is non-negative, with either a half-normal, truncated, or exponential distribution. Likelihood ratio tests were conducted to determine the appropriate distribution, as suggested by Rosko [45] and the exponential distribution was chosen. The two parts of the disturbance, i.e., statistical noise v and productive inefficiency u, should be independently distributed.

From [1], the efficiency (E) of physician i can be defined as:

2 $$ {E}_i=\frac{q_i}{ \exp \left({\mathrm{x}}_i^{\hbox{'}}\upbeta \kern0.5em +\kern0.5em {v}_i\right)}=\frac{ \exp \left({\mathrm{x}}_i^{\hbox{'}}\upbeta \kern0.5em +\kern0.5em {v}_i-{u}_i\right)}{ \exp \left({\mathrm{x}}_i^{\hbox{'}}\upbeta \kern0.5em +\kern0.5em {v}_i\right)}= \exp \left(-{u}_i\right) $$

The E of a physician corresponds to the quantity produced (number of patients seen or visits in one year) divided by the expected quantity of output, with the given inputs and the statistical noise.

Statistical analyses were conducted with STATA© version 13. This study received approval from the Research Ethics Board of the University of Toronto.

## Results

Table 1 shows the descriptive statistics for the sample overall and for the primary care models. Out of the 183 primary care physicians participating in the survey, ten were removed because of missing or very limited data on visits (<10 measured visits for the physician). Half of the physicians excluded were from salaried models and specifically Community Health Centres (CHCs). CHC physicians do not directly bill OHIP and claims records are not generated which reduced the number of salaried physicians from 11 to 6. In addition, 8 observations were excluded because participants did not answer all the questions in the survey, and the result was missing data for some of the study variables. The number of observations for which data were available is indicated for each variable and primary care model in Table 1. The model specification for the SFA was adjusted by removing variables with substantial missing data (variable on FTE counts of providers in the physician practice), and the final statistical model included 165 physicians.

**Table 1:** Descriptive statistics for the sample overall and by primary care model

		All		FFS		FHG		Blended Capitation		Salaried		FHT		Excluded physicians
Variable Name	n	Mean (sd)	n	Mean (sd)	n	Mean (sd)	n	Mean (sd)	n	Mean (sd)	n	Mean (sd)	n	Mean (sd)
*Dependent Variables*														
# of patients seen	174	1,736 (1,118)	17	1,580 (1,292)	40	2,321 (1,741)	54	1,586 (752)	6	1,007 (569)	56	1,564 (646)	9	1,873 (1,499)
# of physician visits	174	5,105 (2,845)	17	5,709 (3,768)	40	6,581 (3,783)	54	4,778 (2,155)	6	3,216 (2,327)	56	4,340 (1939)	9	5,593 (3,707)
Estimated panel ^a^	178	1,636 (1,385)	21	1,914 (1,501)	40	1,899 (1,311)	53	1,371 (617)	10	929 (281)	54	1,762 (1912)	16	2,101 (3,495)
*Inputs*														
Weekly hours	181	40.4 (11.3)	22	44.6 (12.8)	40	42.4 (12.7)	54	37.7 (9.0)	9	38.3 (5.3)	54	38.9 (11.2)	18	38.1 (14.0)
Hours of direct care	181	32.5 (10.6)	22	35.4 (8.8)	40	32.9 (12.9)	53	30.2* (10.0)	10	32.7 (5.4)	55	32.3 (9.7)	16	33.2 (12.1)
Average regular consult duration	183	14.7 (4.9)	22	16.4 (7.0)	40	13.5 (4.8)	54	13.4* (3.7)	10	21.8* (6.2)	56	14.4 (3.6)	18	18.2 (7.1)
Average long consult duration	182	31.3 (9.4)	22	34.9 (12.7)	39	28.8* (9.3)	54	29.6* (7.8)	10	41.5 (11.1)	56	31.0 (8.3)	16	34.6 (14.6)
Percentage of long consult	181	18.0 (10.9)	21	16.0 (12.1)	39	20.5 (11.9)	54	16.9 (8.3)	10	19.0 (18.8)	53	18.2 (9.8)	13	17.8 (14.0)
Percent rural practices	171	12.0	14	18.2	39	5.1	52	5.6*	10	50.0	54	8.9	13	12.5
# of consult rooms	183	21.1 (10.2)	22	19.2 (9.8)	40	22.2 (9.9)	54	22.8 (9.7)	10	24.1 (10.8)	56	18.9 (10.6)	18	20.9 (9.0)
*Patient characteristics*														
Average ACG® weight	174	0.795 (0.252)	17	0.898 (0.377)	40	0.738* (0.174)	54	0.802 (0.214)	6	1.119 (0.759)	56	0.766* (0.156)	9	0.834 (0.201)
% patients female	174	58.9	17	55.9	40	59.6	54	59.6	6	55.6	56	58.8	9	59.2
Average patient age	174	42.8 (6.7)	17	45.9 (8.5)	40	39.7** (5.9)	54	43.3 (5.5)	6	49.6 (11.2)	56	43.4 (6.3)	9	43.6 (9.8)
Average income quintile^b^	174	3.1 (0.5)	17	3.0 (0.5)	40	3.0 (0.5)	54	3.2 (0.4)	6	2.5 (0.6)	56	3.1 (0.5)	9	3.2 (0.5)
Percent IQ 1	174	17.6	17	20.7	40	19.2	54	16.3	6	28.9	56	18.2	9	13.9
Percent IQ 2	174	18.8	17	18.5	40	19.0	54	17.8	6	26.4	56	18.5	9	24.1
Percent IQ 3	174	19.0	17	18.4	40	20.2	54	18.9	6	14.6	56	19.1	9	16.6
Percent IQ 4	174	22.1	17	21.2	40	22.3	54	22.2	6	20.1	56	21.6	9	19.8
Percent IQ 5	174	22.1	17	19.6	40	19.1	54	24.5	6	7.8 **	56	22.1	9	25.6

Indicates a significant difference compared to FFS at *p < 0.05; **p < 0.01

^a^The estimated panel was self-reported by physicians in the QUALICOPC survey. The correlation with the number of patients seen and the number of visits indicated was examined and showed low correlation (0.41). A separate SFA was conducted using the estimated panel to test as the output, and the results were consistent

^b^Various ways of adjusting for the socio-economic status of patients were tested. The results were consistent across specifications, and the average income quintile was selected to limit the number of explanatory variables. The distribution is reported in this descriptive table in order to provide more specific information about the characteristics of the patients in each model

Mean efficiency scores for all physicians and according to primary care model are reported in Table 2. The average efficiency score was 0.722 when using the number of visits as the output and 0.724 when using the number of patients seen as the output. On average, primary care physicians are operating at about 72 % efficiency for each output measure, with a wide variation in the scores from 4 % to 93 % for the visits and 5 % to 94 % for the number of patients seen. One of the lowest efficiency scores was of one salaried physician who had 45 visits and who was identified as an outlier when examining physician outputs prior to conducting the SFA. However, this outlier could not be excluded from the reported analyses because ICES requires a minimum of 6 units and excluding it would have brought the number of salaried physicians to 5. However, analyses were conducted without the outlier physician. Removing the outlier did affect the results in that the coefficients on the salaried physicians changed and became insignificant on the models for each of the outputs (Table 3). Efficiency analyses can be substantively affected by the presence of outliers, which is why we tested the sensitivity of our results to outliers.

**Table 2:** Efficiency scores using an exponential distribution

Variable name	Efficiency- visits		Efficiency- patients seen	
	Mean Efficiency (sd)	min-max	Mean Efficiency (sd)	min-max
All (165)	0.722 (0.182)	0.042 - 0.933	0.724 (0.168)	0.046- 0.936
FFS (16)	0.632 (0.308)	0.042 - 0.887	0.611 (0.282)	0.054 - 0.893
FHG (38)	0.736 (0.162)	0.279 - 0.913	0.707 (0.162)	0.340 - 0.921
Blended Capitation (53)	0.738 (0.124)	0.419 - 0.933	0.752 (0.109)	0.387 - 0.936
Salaried (6)	0.647 (0.326)	0.062 - 0.874	0.674 (0.314)	0.046 - 0.873
FHT (52)	0.740 (0.163)	0.046 - 0.929	0.738 (0.155)	0.126 – 0.912

**Table 3:** SFA results with an exponential distribution

Variable name/output	Number of visits	Number of patients seen
*N* = 165		
FFS - reference		
FHG	−0.054	−0.053
Blended Capitation	−0.228^b^	−0.191^c^
Salaried Models	−0.310	−0.372^c^
FHT	−0.255^a^	−0.084
Rural	−0.133	−0.066
Ln(hours spent on direct care)	0.213^a^	0.187^b^
*Patient Characteristics*		
Ln (average income quintile)	−0.446^c^	−0.461^c^
Ln(average age)	0.166	−1.133^a^
Ln(percent female)	−0.545^c^	−0.408
Ln(average ACG)	0.062	0.151
*Consult Time*		
Ln(Percent long consult)	−0.246^a^	−0.214^a^
Ln (long consult time)	0.003	−0.360^c^
Ln(regular consult time)	−0.852^a^	−0.377^c^

Coefficient significant at: ^a^ < 0.001; ^b^ < 0.01; ^c^ < 0.05

Table 3 reports the results from the SFA, using the number of visits in the first column and the number of patients seen in the second column. Analyses were run with other model specifications that included more variables such as the number of consultation rooms, different measures of patient health status and socioeconomic status, and the results were consistent.

Because the choice of the distribution of the error term can be arbitrary, each of the three common distributions used for SFA models, i.e., half-normal (which is the default), exponential, and truncated was run. They were tested with the likelihood-ratio test to select the distribution with the better fit, and the exponential distribution was selected.

The first analysis used the number of visits as the output. In this model, the coefficients on the primary care model variables show that the number of visits is significantly lower for physicians in blended capitation models and interdisciplinary teams (22.8 % and 25.5 % respectively), as compared to FFS physicians, controlling for patient characteristics and location. The other variables that were significantly associated with lower productivity were: the longer mean duration of a regular consultation and the proportion of long consultations, a higher average income quintile of the patient population, and a higher percentage of females in the physician’s patient population. The average duration of a long consultation was not significant.

Similar results were found when using the number of patients seen as the output. In this case, physicians paid on the basis of blended capitation and salary had a lower output, and the physicians from the other models, i.e., FHG and FHT, had outputs that were not significantly different from those of FFS physicians. Longer durations of a regular consultation and of a long consultation and a higher percentage of long consultations were associated with lower levels of output. The number of patients seen decreased with a higher average age of patients and with a higher average income quintile of patients.

## Discussion

The purpose of this study was to examine the effect of the primary care models on the efficiency of physicians. Efficiency is largely driven by the quantity of services produced by physicians but also determined by the approach to measure the inputs and the outputs, as well as other factors that may affect the quantities of outputs produced.

As expected, the number of visits and the number of patients seen were higher amongst FFS and FHG physicians (Table 1). These results are consistent with the theoretical and empirical literature [7, 47–49]. As mentioned earlier, physician paid through FFS have an incentive to conduct more visits to generate higher income and they can choose to do so by increasing the hours that they spent on direct patient care [9] and/or decrease the time spent per visit [10].

The framework that we developed for this study controlled for some differences in the practice style of physicians and notably, the duration of the visits, and the time that is actually spent on direct patient care. Some of these differences did follow, to a certain extent, the primary care model. For instance, capitated physicians spent fewer hours on direct patient care compared to FFS physicians. FFS physicians worked the longest hours, including the hours spent on direct patient care, which is consistent with evidence that FFS physicians work longer hours [50]. The payment mechanisms may drive physicians to adopt specific practice styles. It may also be that physicians select to practice in a primary care model where the payment incentives align with their preferred practice style. Many observed differences were not statistically significant, likely because of the small sample size. Even though not significant, there are differences between FFS and FHG physicians which are surprising, given the similarities in the payments, as most of the remuneration to FHGs come from FFS payments. FHG physicians serve significantly healthier and younger patients than FFS physicians. FHG physicians also have visit durations that are similar to those of capitated physicians and shorter than FFS physicians. Only salaried physicians have longer visits. Salaried physicians serve patients in poorer health and from lower socio-economic status (28.9 % in the lowest income quintile and only 7.8 % in the highest income quintile).

Physicians may have different resources. We examined the survey data on the FTE counts of administrative staff (manager, medical secretaries) as well as health care providers (nurses, social workers, etc.) and found that the differences followed the primary care models as expected in that FHTs had more employed staff overall. We did not observe a relationship between the duration of the visits and the number of employees. FFS and FHG physicians had the lowest number of other providers. A sensitivity analysis with the FTE counts of the employee types was conducted and no added variables were significant. However, there was a lower response on these variables, resulting in a reduced sample size.

In the efficiency analyses, the differences in the hours spent on direct care and the duration of the visits are controlled for. The results show that the efficiency varied greatly among the sample of primary care physicians (5 % to 94 % efficient). Despite having higher numbers of visits, physicians paid though FFS (including FHG) had lower average efficiency scores than capitated physicians and physicians working in FHTs. FFS physicians had on average the lowest efficiency scores when controlling for confounders.

Although our results are different from those of a previous study on efficiency of primary care physicians in Ontario [21], the difference can be explained by the different methodology. Milliken et al. [21] used data reported from physicians instead of administrative databases for the outputs and for patient characteristics and they did not include any data on the duration of consultations, and they used FTE counts instead of the numbers of hours spent on direct patient care for the physician inputs.

Our results are aligned with those from Gaynor & Pauly [9]. In the model that Gaynor & Pauly developed and empirically tested, financial incentives increased the productivity of physicians in terms of the number of services provided, but the effect was achieved through physicians’ greater effort (i.e., working longer hours). The financial incentives did not affect the efficiency of physicians, when adjusting for effort, by using the number of hours spent on direct patient care.

From a policy perspective, one would need to consider what the desired outcomes of care are. A public health insurer such as the Ontario government aims to provide access to comprehensive care to all. Although FFS physicians may not be more efficient, they do provide more services by working longer hours. The higher numbers of visits in FFS and FHG physicians could be desirable if they reflected higher access to services. However, these numbers may also reflect unnecessary visits that could be substituted with other forms of care. For example, renewal of some prescriptions can be done electronically, and patients could receive a phone call after a test to book an appointment only when results need to be discussed with the physician, avoiding visits for normal test results. One of the limitations in this study is that the appropriateness of the visits could not be measured. Physicians on blended capitation and in FHT had fewer visits, but the numbers of patients seen were similar to those of FFS physicians and their efficiency average scores were higher, which suggests that they may be substituting some visits with for example phone calls (not captured in the outputs in this study) or services with other non-medical providers such as nurses or medical secretaries that they employ. In the salaried models, physicians had fewer visits and served fewer patients. However, the durations of the visits were also longer. Longer visit durations may be necessary to deliver comprehensive care to patients with higher needs. Patients of salaried physicians seemed to have higher needs, with lower health status and lower socioeconomic status.

We were unable to identify a comprehensive measure of quality in our databases and therefore quality of primary care was not directly assessed in the present study. Some studies on efficiency of primary care physicians did have indicators of quality and found a trade-off between quality and efficiency [51]. Although it cannot be assumed that better quality of care requires more time [52–55], a number of studies found a positive association between longer visits and various aspects of the quality of care such as higher patient satisfaction [56, 57] and with higher patient participation in decision making [58]. Longer visits have also been associated with higher provision of preventive services, higher levels of health education, and higher likelihood of screening [57, 59–62]. Hence, adjusting for visit duration in our study is highly relevant as longer visits may in part reflect the delivery of more comprehensive services within the visit, and may be comparable to having multiple shorter visits.

A limitation of the study is the self-selection of physicians into the QUALICOPC study. The distribution across models in our sample is quite similar, albeit with a higher proportion of physicians from FHTs (31 % in our sample vs 22 % in the overall population of primary care physicians) and a lower proportion of physicians in blended capitation models (30 % in our sample vs 42 %) [41]. In terms of socio-demographic characteristics, the physician sample was somewhat different from the Ontario physician population in terms of proportion who were female (56.1 % in our study vs 37.5 %), mean age (49.3 vs 51.1), and being foreign-trained (19.7 % vs 28.3 %). The patient characteristics by model were similar to what others have found for the overall Ontario population [63]. We also examined whether physician characteristics differed across models in terms of age and gender and there were no statistical differences.

## Conclusion

The efficiency scores reported in this study, with an average of 0.72, can be considered low and raise questions about possible avenues to increase the efficiency of physicians in the delivery of primary care. Overall the efficiency scores were relatively similar across the primary care models and for the two output measures. However, there was a high variation within each model and overall, which also indicates that there are gains in efficiency to be made across all physician practice models. Primary care have other objectives than maximizing the volume of services and future efficiency analyses should aim to better measure the marginal product of various forms of physician effort (such as coordinating care with other providers, or phone calls and email communications) on patients’ access to primary care services and on patient health.

## Abbreviations

ACG®, adjusted clinical group; CHC, community health centres; DAD, discharge abstract database; FFS, fee-for-service; FHG, family health group; FHT, family health team; FTE, full time equivalent; ICES, institute for Clinical Evaluative Sciences; IKN, ICES key number; MOHLTC, ministry of health and long term care; NACRS, National ambulatory care reporting system; OHIP, Ontario health insurance plan; QUALICOPC, quality and cost of primary care sudy; SFA, stochastic frontier analysis; SoB, schedule of benefits.
